# Focal Therapy in the Management of Prostate Cancer: An Emerging Approach for Localized Prostate Cancer

**DOI:** 10.1155/2012/391437

**Published:** 2012-04-24

**Authors:** Takeo Nomura, Hiromitsu Mimata

**Affiliations:** Department of Urology, Oita University Faculty of Medicine, 1-1 Idaigaoka, Hasama-machi, Yufu, Oita 879-5593, Japan

## Abstract

A widespread screening with prostate-specific antigen (PSA) has led increased diagnosis of localized prostate cancer along with a reduction in the proportion of advanced-stage disease at diagnosis. Over the past decade, interest in focal therapy as a less morbid option for the treatment of localized low-risk prostate cancer has recently been renewed due to downward stage migration. Focal therapy stands midway between active surveillance and radical treatments, combining minimal morbidity with cancer control. Several techniques of focal therapy have potential for isolated ablation of a tumor focus with sparing of uninvolved surround tissue demonstrating excellent short-term cancer control and a favorable patient's quality of life. However, to date, tissue ablation has mostly used for near-whole prostate gland ablation without taking advantage of accompanying the technological capabilities. The available ablative technologies include cryotherapy, high-intensity focused ultrasound (HIFU), and vascular-targeted photodynamic therapy (VTP). Despite the interest in focal therapy, this technology has not yet been a well-established procedure nor provided sufficient data, because of the lack of randomized trial comparing the efficacy and morbidity of the standard treatment options. In this paper we briefly summarize the recent data regarding focal therapy for prostate cancer and these new therapeutic modalities.

## 1. Introduction

Prostate cancer is one of the most common cancers in the developed countries [1]. Statistically it has overtaken lung and colon cancers to be the most common cancer in male. One of the most important advances in prostate cancer management in recent years is the discovery of prostate-specific antigen (PSA) as a tumor marker [2]. A 75% decrease in metastatic prostate cancer and a 91% increase in localized disease with patients diagnosed annually have been observed since 2002 [3]. That is, the PSA screening has resulted in an increased detection rate of prostate cancer with stage migration towards lower stage, leading to overdetection and overtreatment of prostate cancer by at least 30% [4, 5]. A dramatic diagnostic paradigm shift has forced urologists to reevaluate the role of traditional radical therapies such as external beam radiotherapy and prostatectomy. Maintaining quality of life is as important as prostate cancer eradication. Thus, it is no longer acceptable to just cure prostate cancer patients by aggressive treatments in downward grade and stage migration as well as declining age of prostate cancer diagnosis. Because radical treatments carry significant morbidity with operative complications (hemorrhage, pain, etc.) and long-term toxicity (incontinence, impotence, rectal problems, etc.), there has been a great need for developing ablative therapies that attempt to reduce treatment burden with assuring good cancer control and avoiding the psychological morbidity associated with active surveillance. In addition, it seems reasonable that interest has been considerable in adapting focal methods because the prostate is easily accessible by way of the rectum, urethra, or perineum. Partial surgery of the prostate is impossible due to the location of the cancer in the periphery of the prostate gland, which to access necessitates the almost same morbidity as removing the whole gland. Therefore, focal therapy using energy modalities offers generally accepted only solution for partial treatment of the prostate gland.

Focal therapy has been introduced as middle ground alternative between active surveillance and radical treatments with effective early cancer cure or control. The terms “focal therapy” and “organ-preserving therapy” may be defined as complete selectively ablation techniques of clinically significant cancer foci within prostate in a focal or subtotal manner with the overall objective of minimizing lifetime morbidity without compromising life expectancy. That is to say, the energy modalities must be easily delivered to the prostate and be capable of destroying cancer cells. The obvious benefit of focal therapy is preservation of the uninvolved surround healthy tissues such as the sphincter, the neurovascular bundles, and normal prostate gland using a minimally invasive technique [6]. And there may be a potential to repeat focal therapy or use another treatment modality in case of persistent cancer. On the other hand, the main issue of prostate cancer is multifocal localization of cancer foci [6]. The patients with unifocal, unilateral, or low volume prostate cancer are most suitable for focal therapy; however, we found a great deal of difficulty in identifying patients with multifocal clinically significant cancer foci who require aggressive whole gland therapy from those with clinically focal cancers who may benefit from organ-sparing treatment. It has been reported that the oncological outcome was similar between the unilateral or bilateral cancer groups in patients with low-risk prostate cancer, suggesting that the limiting factor for focal therapy is clinical risk stratification, not laterality of cancer [7]. Indeed prostate cancer has long been recognized as characteristically multifocal, but it may present as true unifocal or volume-limited multifocal disease in the era of widespread PSA screening and early detection. Therefore, improved imaging techniques and mapping biopsy protocols in patient selection are needed to fully support focal therapy.

In this paper we briefly discuss the evidence for a variety of ablative energy modalities available for use in focal therapy of localized prostate cancer including cryotherapy, high-intensity focused ultrasound (HIFU), and vascular-targeted photodynamic therapy (VTP).

## 2. Cryotherapy

The first report describing cryotherapy of benign prostate hyperplasia appeared in 1966 [8], and an attempt to destroy prostate cancer by using a transperineally introduced cryoprobe was reported in 1972 [9]. Although cryotherapy did not achieve wide usage initially due to incomplete eradication of the tumor or high recurrent rate of cancer [10, 11] and high complication rates including urinary retention, incontinence, urethrorectal or urethrocutaneous fistulas, stricture, chronic rectal or perineal pain, and loss of erections, advantages of the procedure were recognized [12]. In particular, use of transrectal ultrasound (TRUS) for real-time monitoring of the freezing process, improved cryoprobe system, and a urethral warmer using a continuous irrigation system could popularize cryotherapy as an effective and technically feasible treatment for prostate cancer [13]. Finally, cryotherapy was approved as treatment for prostate cancer by Centers for Medicine and Medical Services in 1999 [14].

Cryotherapy is the localized destruction of tissue by low temperature and thawing, which causes direct injury to cells as well as secondary injury from the inflammatory response of the body. Current technology uses argon gas or liquid nitrogen circulating through hollow needles to freeze the prostate and helium gas to warm the urethra via the Joule-Thompson effect. There are three treatment parameters correlated with cancer cell destruction: the cooling rate, the lower temperature, and the duration of the freeze cycle. Complete cell death is likely to occur at temperatures lower than −40°C for two cycles. After reaching a tissue temperature of less than 0°C, the extracellular fluid starts to crystallize and formation of crystals causes hyperosmotic pressure of the unfrozen portion of the extracellular fluid compartment, leading to water shifting from the intracellular space to the extracellular space. The cell water loss induces intracellular dehydration and pH change. This is followed by cell shrinkage and denaturing of cellular proteins. With further drops in temperature less than −15°C intracellular crystallization takes place and cell metabolism begins to fail. This mechanically breaks the cellular membrane and the cell apoptosis is also induced after thermal injury. The apoptotic cells are observed primarily in the peripheral zone of the cryogenic lesion outside the killing zone, where the temperature is not fully decreased to kill all the cells [15]. As temperatures rise, extracellular fluid shifts back again into the intracellular space, leading to cellular bursting. The vasodilation around the targeted tissue occurs after thawing causing hyperpermeability of the vessel wall. This leads to endothelial damage and microthrombi formation resulting in regional tissue hypoxia and secondary necrosis of the tissue [14, 16].

A significant current development is the introduction of cryotherapy probes that use argon gas rather than liquid nitrogen. Argon gas can rapidly cool the probe tip to −187°C and can be rapidly exchanged with helium gas at 67°C for an active thawing phase, producing a faster response to operator input and significantly speeding 2-cycle treatment [17]. Moreover, argon-based probes have a much smaller diameter. Thus, they permit direct, sharp transperineal insertion using transperineal placement of ultrathin probes through a brachytherapy template, avoiding the need for tract dilation and facilitating more conformal cryosurgery by allowing placement of more probes [18].

## 3. Patient Selection

This modality can be used in any tumor grade of prostate cancer with clinical stage T1c-T3 disease. In general, primary cryotherapy is suitable for patients with low-risk group (clinical stage T1c-T2a disease, Gleason grade 6, and PSA < 10 ng/mL who are not potent or not interested in maintaining their potency). Patients with intermediate risk group (Gleason grade 7, PSA between 10 and 20, or clinical T2b) are also effective for the procedure. Because of the minimally invasive therapeutic modality, cryotherapy may have specific advantages in selecting patients with certain comorbidity including those who cannot tolerate radical prostatectomy or radiation therapy (e.g., persons with previous pelvic radiation or pelvic surgery, irritable bowel disease, cardiac disease, extreme obesity) [14]. Contraindication to cryotherapy includes the presence of tumor foci near the urethra because the urethral warming catheter will preclude complete eradication of disease [16]. In addition, this therapy is generally contraindicated in patients with the presence of tumor foci near the neurovascular bundles if patients are potent or interested in maintaining their potency [16]. Patients with severe lower urinary tract symptoms or large prostate are also poor candidates for cryotherapy, and a previous transurethral resection of the prostate (TURP) is generally considered a contraindication. If the prostate size exceeds 50 mL, neoadjuvant hormone therapy is needed to reduce the prostate volume because complete freezing of the prostate is difficult [14, 19]. However, there is no evidence whether neoadjuvant therapy or combination therapy with androgen deprivation influences postcryotherapy cancer control [14].

## 4. Clinical Outcomes

There were a lack of consensus on how recurrence was defined and no accepted biochemical definition of PSA failure after primary cryotherapy. The PSA value rises immediately after cryotherapy due to release of intracellular PSA from cellular necrosis and PSA nadir is usually achieved more than 3 months after the procedure [20]. Serum PSA levels are unlikely to reach undetectable levels because some PSA-producing periurethral prostatic tissue will remain. Commonly, in order to assess local control patients who have been treated with cryotherapy have undergone repeat prostate biopsy 6 to 12 months after cryotherapy in several series. The positive biopsy rate after the procedure ranges from 7.7% to 23% [13, 19, 21, 22]. These studies are including focal targeted cryoablation, hemiablation, and radical cryoablation. Higher initial PSA levels and clinical T stage were positively associated with a risk of positive biopsy rate after cryotherapy. In 860 patients treated with focal cryotherapy included in the Cryo On-Line Data (COLD) Registry, 5-year biochemical disease-free rates ranged from 77.6% to 82.4% according to the American Society for Therapeutic Radiology and Oncology (ASTRO) criteria of three consecutive PSA rises after the posttreatment nadir and 58.0% to 74.9% according to the Phoenix criteria of PSA nadir plus 2. For this series, 21% had evidence of cancer at postcryotherapy biopsy [23]. Taken together, results of recent reports indicate that 5-year biochemical disease-free survival rates range from 60% to 90% for patients with low- and intermediate-risk groups [24–27]. A recent randomized trial to compare external beam radiotherapy to cryotherapy for patients with localized prostate cancer receiving neoadjuvant antiandrogen therapy indicated cryotherapy to be noninferior to external beam radiotherapy in disease progression (23.9% in cryotherapy versus 23.7% in radiotherapy) at 36 months and 5 years overall (89.7% versus 88.5%) and disease-specific survival at 5 years (96.4% versus 96.1%) [28].

## 5. Complications

Erectile dysfunction and impotence after cryotherapy are common. Some series have reported impotence rates ranging from 50% to 92% [27, 29]. This is probably because of the use of multiple freeze-thaw cycles and the extension of the damage beyond the prostate, into the area of the neurovascular bundles; however, complete ablation of the neurovascular bundles is necessary to ensure eradication of cancer at the periphery of the prostate. Interestingly, impotence rates after hemiablation ranged from 10% to 29% and potency made a recovery within 1 year, although subtotal or focal cryotherapy should be investigational [30, 31]. A recent randomized trial reporting on quality of life outcomes showed that cryotherapy was associated with poorer sexual function comparing with external beam radiotherapy. Patients who wish to increase their odds of retaining sexual function may be better to choose other modalities over cryotherapy [32]. Cryotherapy is associated with more acute urinary dysfunction, which resolves over time [32]. The reported incidence of urinary incontinence ranges from 3.7% to 4.8% [26, 27, 29]. The causes of incontinence include sphincter muscle destruction, disruption of the pudendal nerve, and urethral sloughing.

There are some complications specific to cryotherapy including tissue sloughing, urethral stricture, pelvic and rectal pain, rectourethral fistula [33], and penile numbness [19]. Penile and scrotal swelling within several weeks after cryotherapy is reported as a rare complication. Hydronephrosis or small bowel obstruction as uncommon complications results from the extensive freezing and deep insertion of cryoprobe [34]. The risks of these complications have decreased with advances in technology, such as urethral warming techniques, and improved patient selection.

## 6. High-Intensity-Focused Ultrasound (HIFU)

The first report describing HIFU using the transrectal probe of benign prostate hyperplasia appeared in the mid-1980s [35], and an attempt to treat localized prostate cancer was reported in 1995 [36]. Diagnostic ultrasound usually uses frequencies in the range of 1 to 20 MHz, but transrectal HIFU uses sound waves with frequencies of 0.8 to 3.5 MHz and can achieve coagulative necrosis and the destruction of the targeted tissue through hyperthermia in two ways. The two mechanisms of tissue damage are by the conversion of mechanical energy into heat and inertial cavitation. Firstly, ultrasound energy is concentrated and tissue absorption of the focused ultrasound wave generates temperatures that exceed 80°C, which denature proteins and destroy lipid-based membranes, and this process finally results in instantaneous and irreversible coagulative necrosis. Secondly, the alternating cycles of compression and rarefaction develop inertial cavitation effect causing additional damage to the prostate and periprostatic tissue. Histologically, homogeneous coagulative necrosis developed in the damaged tissue with an inflammatory response that follows leading to formation of granulation [37]. Currently, two systems including Sonoblate-500 produced by Focus Surgery (Indianapolis, IN) and Ablatherm produced by EDAP TMS (Lyons, France) for delivery of HIFU are available. The treatment area is heated for 3 seconds and cooled for 6 seconds with real time images. The energy decreases sharply outside the target zone; thus the surrounding tissues are minimally affected. Days to months are required for necrosis and cavitation to occur, and the prostate gland shrinks a small size over 3 to 6 months after the procedure. Due to the limits of the ultrasound wave, there is the difficulty in ablating the whole prostate gland, especially in a large prostate (>40 mL) and the difficulty in ablating anterior cancers [38]. Hormone therapy or transurethral resection of the transitional zone may be useful in overcoming the difficulty of reaching the anterior zone.

## 7. Patient Selection

In general, primary HIFU is suitable for older patients (over 70 years) with low- and intermediate risk groups. HIFU may be used to treat prostate cancer, either as a primary or as salvage therapy. There is an upper limit to prostate gland size of 40 mL due to focal length of the probe. In this case, a TURP before HIFU can be of benefit to reduce prostate size and can also reduce morbidity and indwelling catheter time. In general, the prostate with highly calcifications (>1 cm) should be contraindication because these will obscure ultrasonographic visualization. In some cases TUR of large calcifications may be performed before HIFU. As with cryotherapy, HIFU may have specific advantages in selecting patients with certain comorbidity including those who cannot tolerate radical prostatectomy or radiation therapy.

## 8. Clinical Outcomes

As the data on HIFU as salvage therapy were limited, we focused on HIFU as primary therapy. The combination of PSA value and prostate biopsy is used to define recurrence after HIFU. A multicenter trial with the results of using the Ablatherm was reported in 2003 [39]. Although 28% of the patients required two treatment sessions, 87% of the patients had a negative biopsy with 92%, 86%, and 82% in the low-, intermediate-, and high-risk groups, respectively. Mean PSA nadir in the low-, intermediate-, and high-risk groups was 1.3 ng/mL, 1.4 ng/mL, and 3.1 ng/mL, respectively, and a median PSA nadir of 0.4 ng/mL was achieved at a minimal follow-up of 6 months. Gelet et al. reported the long-term results in patients with low-risk disease. Patients with preoperative PSA less than 10 ng/mL demonstrated negative biopsy of 78% and 5-year disease-free survivals of 83%. For those with intermediate- and high-risk groups, the disease-free survival rate was 53% and 36%, respectively [40]. Blana et al. also analyzed a large cohort in patients with low- and intermediate- risk disease. The 5-year disease-free probability was 66%, and 28% of the low-risk disease developed treatment failure [41]. The negative biopsy rate after the procedure ranges from 55% to 100% in recent trials including clinical T1 to T3 stages. Higher clinical T stage and larger prostate size were positively associated with a risk of both PSA failure and positive biopsy rate after HIFU [39, 42–44].

## 9. Complications

Transient urinary retention arising from swelling of the prostate after HIFU is the common complication, which may require prolonged catheterization or cystostomy drainage [39]. Prolonged retention ranged from 6% to 32% [39, 42–44]. A high degree of urethral stenosis near the verumontanum and bladder outlet obstruction was seen, which are late complications of HIFU. The recent reported incidence of urethral stenosis ranged from 2% to 17% [39, 42–44]. De novo erectile dysfunction and impotence were also known to occur at 24%–77% of those who were potent preoperatively. The use of a pulsed-wave Doppler ultrasound system to visualize the neurovascular bundles during treatment may improve this outcome. Mild to moderate stress incontinence rates after HIFU ranged from 6% to 14% of patients in earlier series, but this has decreased over the years with next-generation HIFU devices [39, 42–44]. Severe incontinence developed in 1%–5% requiring intervention [39, 42–44]. Stress incontinence, urethral stenosis, and bladder outlet obstruction were reported to be decreased by TURP before HIFU [45]. Therefore, TURP may be indicated before HIFU; however, TURP appears to have no effect on cancer control including PSA nadir, negative biopsy rate, and biochemical failure. There were some serious complications specific to HIFU including rectal wall burn and rectourethral fistula before the use of the rectal cooling device and robotic control system of rectal distance [46]. Current results report on the complications seen with HIFU as whole gland therapy; however, one can expect lower rates of complications with HIFU as targeted focal therapy.

## 10. Vascular-Targeted Photodynamic Therapy (VTP)

VTP is an investigational ablative technology which employs the use of photosensitizing properties that is selectively taken up by prostate cancer cells and produces radical oxygen species upon exposure to light of a specific wavelength which results in the destruction of the tissue. The first report describing photodynamic therapy for prostate cancer with light-sensitive agent (either hematoporphyrin derivative or polyporphyrin photofrin) using a transurethral approach appeared in 1990 [47]. The photosensitizers have taken a long time to be cleared from the body and accumulated in the skin. To avoid sunburn-like reaction patients must have been covered from sunlight for a several weeks after treatment [48]. Recent advances in photodynamic therapy have led to improvements of the synthesis of new generation photosensitizers with more excellent stability and shorter half-lives with faster metabolism. The rapid clearance of these new agents from the circulation and then from the liver could negate the need to avoid exposure to sunlight for long periods. Vascular acting photosensitizers currently under investigation are Tookad (WST09: padoporfin; palladium bacteriopheophorbide) and its water-soluble derivative WST11 (padeliporfin; palladium bacteriopheophorbide monolysotaurine) produced by Steba Biotech (The Netherlands), which are the most widely used new generation photosensitizers. Both WST09 and WST11 remain confined to the vascular bed [49].

A photosensitizer is injected intravenously and is distributed throughout the body during treatment. Under ultrasound guidance, small energy-delivering probes can be positioned in the prostate using a needle placement grid developed for brachytherapy. VTP usually uses an intravenously administered WST09 that absorbs light in the visible near infrared wavelength with maximum light energy absorption at 763 nm. This long light absorption wavelength allows for a deeper light penetration into tissues. A photosensitizer enhances sensitivity of the tumor vasculature to light energy. Damage to the vascular endothelium is followed by platelet aggregation and vascular coagulation round the tip of the fiber with subsequent localized tissue necrosis [50].

## 11. Clinical Outcomes

A first multicenter phase I/II clinical trial reported the safety and efficacy of VTP using WST09 in patients with recurrent localized prostate cancer after external beam radiotherapy including clinical T1 and T2 stages. VTP-treated lesions were generally ellipsoidal on MRI and repeat target biopsies at 6 months, which correlated with devascularized zones on MRI, showed fibrosis, and were devoid of cancer [51–53]. In contrast, no effective areas on MRI included the presence of cancer. Since VTP did not attempt to ablate whole prostate gland in this trial, the PSA changes only reflect the destruction of prostate tissue and not necessarily of prostate cancer. There were no serious adverse events including cutaneous photosensitivity, and neither urinary nor erectile function was compromised in the long-term follow-up up to 6 months after the procedure. In general, VTP using WST09 was performed safely and well tolerated with no serious adverse events including incontinence, tissue sloughing, and rectal injury [51]. This safety may be a result of VTP using the vascular targeting nature of WST09 of the limited light exposure of the urethra and rectum. In contrast, a patient with meso-tetra-hydroxyphenyl chlorine (mTHPC, Foscan) developed a rectourethral fistula after assessing abnormal rectal mucosa by a rectal biopsy [54]. And other side effects including urinary retention, temporary stress incontinence, and urethral stricture were noted in VTP using mTHPC [54].

## 12. Up-to-Date Biopsy Technique and Imaging Modality

The success of focal therapy is obviously dependent on the ability to detect the extent of prostate cancer and then accurately target it. The problem is that we have lack of agreement on candidate selection protocols for focal therapy. This point derives from the lack of an adequate biopsy techniques and imaging modality that could reliably detect prostate cancer foci. There is no clear answer as to what is the optimal biopsy strategy to be used to evaluate potential candidates for focal therapy. However, biopsy is the only accurate method for prostate cancer laterality. There are insufficient data on standard sextant biopsy to assure correct localization of prostate cancer foci [55]. Even extended TRUS-guided saturation biopsy has appeared to be inadequate in the proper selection of patients for focal therapy [56]. At present, transperineal 3D mapping biopsy has been proposed as a way to more accurately predict prostate cancer focality [57, 58]. In this approach, samples are taken every 5 mm throughout the volume of the prostate using a brachytherapy template grid under TRUS guidance. Using transperineal 3D mapping biopsy, 61.1% of patients with unilateral cancer on TRUS biopsy were positive bilaterally, and 22.7% were upstaged by Gleason scores [58]. Therefore, transperineal 3D mapping biopsy may provide the most accurate cancer localization information and appear essential for proper patient selection for focal therapy. The chief objection to this type of procedure is time demand and cost involved. Thus, an alternative imaging technology for tumor detection and localization must be considered. MRI is the gold standard over the past decade. Improvements in functional MRI techniques include MR spectroscopy (MRS), diffusion-weighted imaging (DWI), and dynamic contrast enhanced MRI (DCE). Each of these three techniques has improved tumor detection compared with standard T2-weighted imaging (T2WI) alone [59–61]. The combined diagnostic accuracy of T2WI, DCE, and MRS enabled tumor detection with significant independent and additive predictive value [62]. Multiparametric MRI (MpMRI) is currently considered the state of the art for prostate imaging with reasonable sensitivity and specificity values.

## 13. Conclusion

The recent downward stage migration favoring early disease has revealed new treatment options for patients with localized prostate cancer. And the therapeutic dilemma between active surveillance and radical therapy and the significant morbidity associated with radical therapy have led to development of minimally invasive focal therapy such as cryotherapy, HIFU, and VTP. Cryotherapy and HIFU emerged as pioneers in focal therapy showed a lot of promise but still need a long-term follow-up for assessing quality of life and cancer-specific and overall survival before the indications for primary or salvage therapy can be expanded. VTP as an investigational therapy needs careful patient specific planning since significant variability in the dose distribution and consequent tissue response. The goal of focal therapy is to selectively ablate known disease, while minimizing lifetime morbidity without compromising life expectancy. To reduce the side effects and maintain a favorable quality of life after focal treatment, we must pursue alternative techniques such as a subtotal gland treatment, hemiablation, or focal targeted ablation. The ideal patients for focal therapy appear to be ones with low-grade, low-volume disease that can be easily characterized. In addition, focal therapy may be potentially useful in salvage therapy. Since prostate cancer is commonly a multifocal disease, standard or extended biopsy techniques are not capable of reliably identifying all existing cancer. Therefore, the technical innovations such as 3D-mapping prostate biopsy, histoscan, and MpMRI are applied to accurately detect prostate cancer foci. A further novel imaging tool for identifying individuals is desirable in careful preoperative patient selection.
